# Formation of Highly Selective Binding Pockets in UiO‐Type MOFs From Bridged Linkers

**DOI:** 10.1002/anie.5435518

**Published:** 2026-09-10

**Authors:** Tobias Hennig, Erik Rohloff, Verena Drewes, Adrian Hannebauer, Sergi Plana‐Ruiz, Yaşar Krysiak, Andreas M. Schneider, Andreas Schaate

**Affiliations:** ^1^ Institute of Inorganic Chemistry Leibniz University Hannover Hannover Germany; ^2^ Crystallography and Mineralogy Unit Department of Geology Universitat Autònoma de Barcelona Bellaterra Spain; ^3^ Laboratory for Nano and Quantum Engineering Leibniz University Hannover Hannover Germany; ^4^ Cluster of Excellence PhoenixD (Photonics Optics and Engineering – Innovation Across Disciplines) Leibniz University Hannover Hannover Germany

**Keywords:** electron diffraction, metal–organic frameworks, molecular recognition, pore engineering, UIO‐66

## Abstract

Metal–organic frameworks (MOFs) with precisely modified pore environments are essential for applications in catalysis, gas capture, and gas sensing. However, creating these highly selective sites through straightforward synthetic routes remains a challenge. Herein, we present a design strategy for UiO‐type MOFs that enables control over the precise placement of functional groups within the tetrahedral pores of the framework, distinguishing it from less precise post‐synthetic modification methods. By crosslinking two linker molecules with a short functional urea bridge pre‐synthetically, the combination of the resulting linker geometry and the UiO‐66 framework confines the crosslink to the trigonal pore windows. This results in pocket‐like arrangements, as the urea bridges effectively “wall off” some of the pore windows. The structural implications are supported by modelling and electron crystallography. To demonstrate the functional versatility, we probe the selectivity of the obtained MOF toward aliphatic nitro compounds at low concentrations using vapor sorption measurements and compare it with other UiO‐66 derivatives.

## Graphical abstract

Much room for design: We present a generalizable strategy for precisely modifying UiO‐type Metal‐organic frameworks with functional groups in a site‐specific manner. Through the use of *pre‐connected* linkers, pocket‐like pores for molecular recognition are generated. The high affinity of these binding pockets is demonstrated for capturing aliphatic nitro compounds.

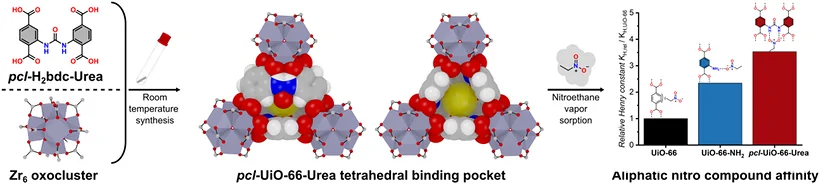

## 1 Introduction

The field of metal–organic frameworks (MOFs) has attracted considerable research interest in the last few decades, not only because of record‐breaking porosities by this class of inorganic–organic hybrid materials, but also because of their highly modular design. By combining inorganic building units (IBUs) and organic linkers with varying coordination geometries, a nearly unlimited variety of network structures can be synthesized [1]. For a given framework type, modification opportunities are also ample: Linker elongation as well as pre‐ and post‐synthetic modification enable tailoring of MOFs [2].

Tailoring the environment of MOF pores through functional groups requires precise control over their steric arrangement. Due to their inorganic–organic nature, distinct reactive sites are defined by the MOF structure itself, which differentiates MOFs from traditional porous materials such as zeolites. Consequently, it has been proposed that MOF pores could be functionalized to mimic proteins, which feature complex binding motifs with well‐defined spatial arrangements of several functional groups [3]. However, general modification strategies providing high control over the steric arrangement of introduced functional groups remain relatively unexplored. Modifying, for example, a terephthalic acid linker at a single position may result in four different possible positions of the functional group within the framework, hindering cooperative effects. Thus, differentiating functional group positions in frameworks composed of a single linker type remains challenging. This complexity is further increased in multivariate MOFs (MOFs composed of several differently modified linkers that share the same length and connectivity), where linker distribution is difficult to probe and may vary between random, alternating, or clustering modes, depending on linker interactions [4].

Precise placement of functional groups is restricted to a very small subset of MOFs that feature several distinct linker positions within a single framework of low symmetry. Seminal work by telfer et al. demonstrated this for the quaternary Zn‐based MUF‐7/77 frameworks, where three crystallographically distinct linkers were incorporated in the same MOF through a one‐pot synthesis procedure. Usually, such an approach results in the co‐crystallization of several different MOF phases. However, subsequent modification of each linker allowed for the construction of pre‐programmed pore environments, enabling control over the regio‐ and stereoselectivity of asymmetric organocatalytic transformations [5, 6, 7].

A similar concept was realized in more stable Zr‐based MOFs by Zhou et al., who intentionally lowered the symmetry of a UiO‐67‐based framework by introducing bulky substituents that force the biphenyl‐4,4′‐dicarboxylate linkers into a perpendicular conformation. The resulting eight‐connected framework featured unoccupied gaps between the IBUs of varying lengths, which were subsequently bridged through post‐synthetic linker installation. Modification of the newly installed linkers allowed for size‐selective oxidation reactions within the MOF pore system [8, 9].

Both concepts, however, lack the versatility required for a more universal design strategy applicable to other MOF systems. We aim to address this gap using pre‐synthetically bridged linkers, which form *oligo*‐ or *pcl*‐ (*pre‐connected linker*) MOF structures. These *pre‐connected linkers* stay spatially associated during framework formation and thus allow for more precise functionalization. This is accomplished by either modifying each linker independently or by using a crosslink that is functional in itself and then choosing crosslink length and geometry carefully. It is important to distinguish this modification strategy clearly from conventional post‐synthetic modification, as the pre‐connected linker strategy retains spatial information imparted on the linker through the pre‐synthetic crosslinking, whereas post‐synthetic modification may only modify the framework with randomly distributed functional groups.

As first shown by Cohen et al., linkers connected by flexible [10] or rigid crosslinks [11] readily form the same parent MOF structure as their non‐connected counterparts, provided certain geometric conditions are met. Beyond simple dimeric linkers, oligomeric linkers featuring up to eight connected linker units [12] and even polymeric linkers [13, 14] may be incorporated into MOF structures. Several advantages of *pcl*‐MOFs have been reported in the literature, including improved hydrolytic stability [15], the prevention of network interpenetration and pore collapse [16], and enhanced gas separation selectivity through tuning the pore window sizes [17, 18]. Furthermore, the synthesis of *pcl*‐MOFs followed by the subsequent decomposition of crosslinks has also been demonstrated, revealing templated functional groups [19]. In a particularly notable series of studies, Xiao et al. successfully templated pairs of carboxylic acids [20], amino groups [21], and even acid–base pairs [22] into an expanded Mg‐MOF‐74 analogue, achieving improved catalytic performance for the latter compared to non‐templated frameworks.

In this work, we present a strategy to template functional groups into UiO‐66 with precise distances from one another. By utilizing the structural constraints of *pre‐connected linkers*, we can introduce local asymmetry into the high‐symmetry framework, enabling site‐specific functionalization. Unlike the emergence of stochastic, long‐range aperiodicity found in Truchet‐tiling MOFs, our method maintains the periodic connectivity of the lattice while controlling local asymmetry [23]. Following these structural considerations, we designed a bridged linker to construct a MOF with specific binding sites for nitro compounds, the effectiveness of which was evaluated using nitroethane as a probe molecule in vapor sorption. We believe our findings are generalizable to many different framework types, paving the way for more precisely tailored MOFs for specialized applications.

## 2 Results and Discussion

### 2.1 Structural Considerations and Linker Design

The modification strategy presented in the following relies solely on geometric considerations, and therefore it should be applicable to any given MOF structure. As a proof of principle, we have selected UiO‐66 as a prototypical 12‐connected Zr‐based framework for the following reasons: First, its high stability toward water and atmospheric conditions, which is desirable for many applications of precisely modified MOFs [24]. Second, the highly symmetric **fcu** topology, which narrows the geometric considerations when designing bridged linkers (as discussed in detail below).

When using such bridged linkers, the crosslink can be located between two linkers in the square base area (Figure 1a) or in the trigonal pore window (Figure 1b) of the octahedral pore of UiO‐66. Both arrangements still result in periodic structures. When bridging the trigonal pore windows, a shorter crosslink distance may be possible by rotating both adjacent linkers out of the carboxylate plane toward each other. This is not possible when bridging across the square window occurs, as both linkers are already within the same plane. More detailed information about the crosslinked structures can be found in Section S5, Structural Considerations.

**FIGURE 1:**
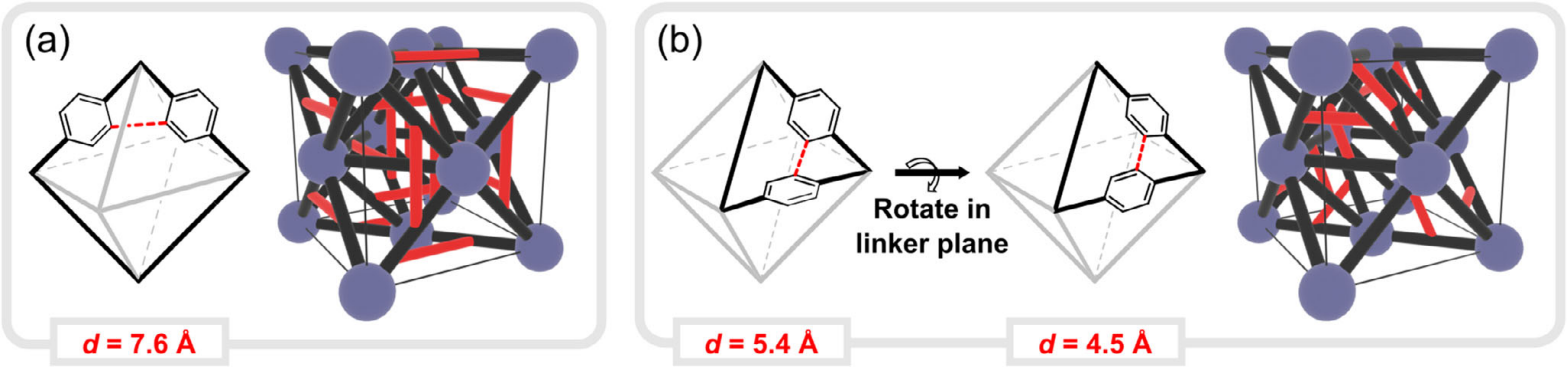
C─C crosslinking distances and resulting periodic models of UiO‐66. The framework is constructed exclusively using bridged linkers. To ensure precise positioning, only distances between phenyl ring carbon atoms nearest to a shared IBU of two linkers are considered. (a) Crosslinking distance and resulting periodic structure when connecting adjacent linkers across the square base area of the octahedral pore. (b) Crosslinking distance and resulting periodic structure when connecting adjacent linkers within the trigonal pore windows. In (b), non‐coplanar linker orientation and rotation in the carboxylate plane result in shorter distances. For a chosen crosslink distance between 4.5 and 5.4 Å, the linker can only be incorporated as depicted in (b). In the periodic structures, linkers spanning the framework are represented by thick black lines, while crosslinks are represented as red solid lines. IBUs are shown as purple spheres. In the schematic octahedral pores, red dashed lines indicate the C─C crosslinking distances of interest. For clarity, IBUs at each corner of the octahedral pores are omitted.

Based on these considerations, we selected a linker with the crosslink spanning the minimum distance the framework can accommodate. This ensures that the crosslink is only incorporated across the trigonal pore windows, resulting in single‐site functionalization. Furthermore, an analytical handle is generated through the aforementioned linker tilting, which could serve to distinguish the resulting crosslinked framework from its parent framework during structural analysis. A potential limitation of this modification strategy is that the amount of chemical information encodable in such a crosslink is limited by the crosslink length in most frameworks. While longer crosslinks can be introduced in principle, this does not guarantee that only a single bridging mode is present within the resulting framework.

For our proof of concept, we opted for a crosslinking element which itself is functional. Both length and functionality requirements are met by employing a urea group as the crosslink. According to the crystal structure of 1,3‐diphenylurea, a C─C distance of around 4.9 Å between the attached phenyl rings is expected, which is slightly shorter than the required 5.4 Å [25]. Therefore, bridging two terephthalic acid linkers via a urea crosslink should yield the desired crosslinked UiO‐66 structure, with linker tilting potentially occurring to accommodate the shorter‐than‐required distance. Analogously, related UiO‐type derivatives like UiO‐67 or UiO‐68 should be modifiable in an identical manner, because the crosslinking distance depicted in Figure 1b is independent of the linker length, provided that the crosslink is introduced at the *ortho*‐position to the coordinating carboxylates sharing an IBU.

The functional versatility of MOFs incorporating urea groups within the linker has been demonstrated in the literature, for example, in gas adsorption [26], heterogeneous catalysis [27, 28, 29, 30, 31], and sensing [27, 28], primarily due to the ability of urea moieties to form strong bifurcated hydrogen bonds to other functionalities such as nitro groups [29]. While most reported M^2+^‐based (Zn^2+^, Cu^2+^, Cd^2+^, Ca^2+^, Mn^2+^) MOFs feature the urea group within the linker backbone, Zr‐based urea‐modified MOFs like UiO‐67 [30] or UiO‐68 [31] rely on introducing functional groups via bulky side chains. In both cases, a mixed linker approach involving unmodified linkers was necessary, as MOFs composed entirely of modified linkers showed significantly reduced porosity. Biswas et al. circumvented this by introducing the urea group through a diazepine motif in the biphenyl‐4,4′‐dicarboxylic acid linker of UiO‐67. However, this results in a cis/cis conformation that likely diminishes hydrogen bonding interactions [32, 33].

### 2.2 Synthesis and Characterization of *Pcl*‐UiO‐66‐Urea

The proposed *pcl*‐H_2_bdc‐Urea linker (see Figure 2a) was obtained on a gram scale through a facile two‐step synthesis (see Section S2, Linker Synthesis, for more detail). To prevent linker rearrangement to the corresponding benzoyleneurea (See Figure S5), a room‐temperature MOF synthesis was adopted. For Zr‐based MOFs, room‐temperature syntheses can be accomplished by pre‐formation of the Zr_6_‐IBU at elevated temperature, which has been reported as early as 2009 by Ferey et al. [34] and has since then been adapted for several other MOFs, allowing for the use of modified linkers and even DMF‐free syntheses [35, 36, 37, 38, 39, 40, 41, 42]. The concept benefits from the ready formation of Zr‐carboxylates at room temperature, whereas the IBU formation requires elevated temperature. We adapted a procedure reported for MOF‐808 by Serre et al. [43] for Zr_6_ cluster synthesis and modified their framework synthesis conditions for *pcl*‐UiO‐66‐Urea. Using a H_2_O/DMF/Acetic acid‐based solvent system together with the *pcl*‐H_2_bdc‐Urea linker and Zr_6_ clusters, *pcl*‐UiO‐66‐Urea nanocrystals could be obtained after stirring the suspension for 7 days at room temperature (See Section S3, MOF Synthesis, for more details). As this synthesis approach is known to produce nanocrystals with high defect concentrations, the de novo synthesized particles were employed as seed crystals to promote the growth of larger particles with significantly less defects. By using these seeds under identical synthesis conditions, we successfully increased the particle size to approximately 200 nm over two growth steps. These enlarged particles proved to be suitable for structural characterization via electron diffraction methods.

**FIGURE 2:**
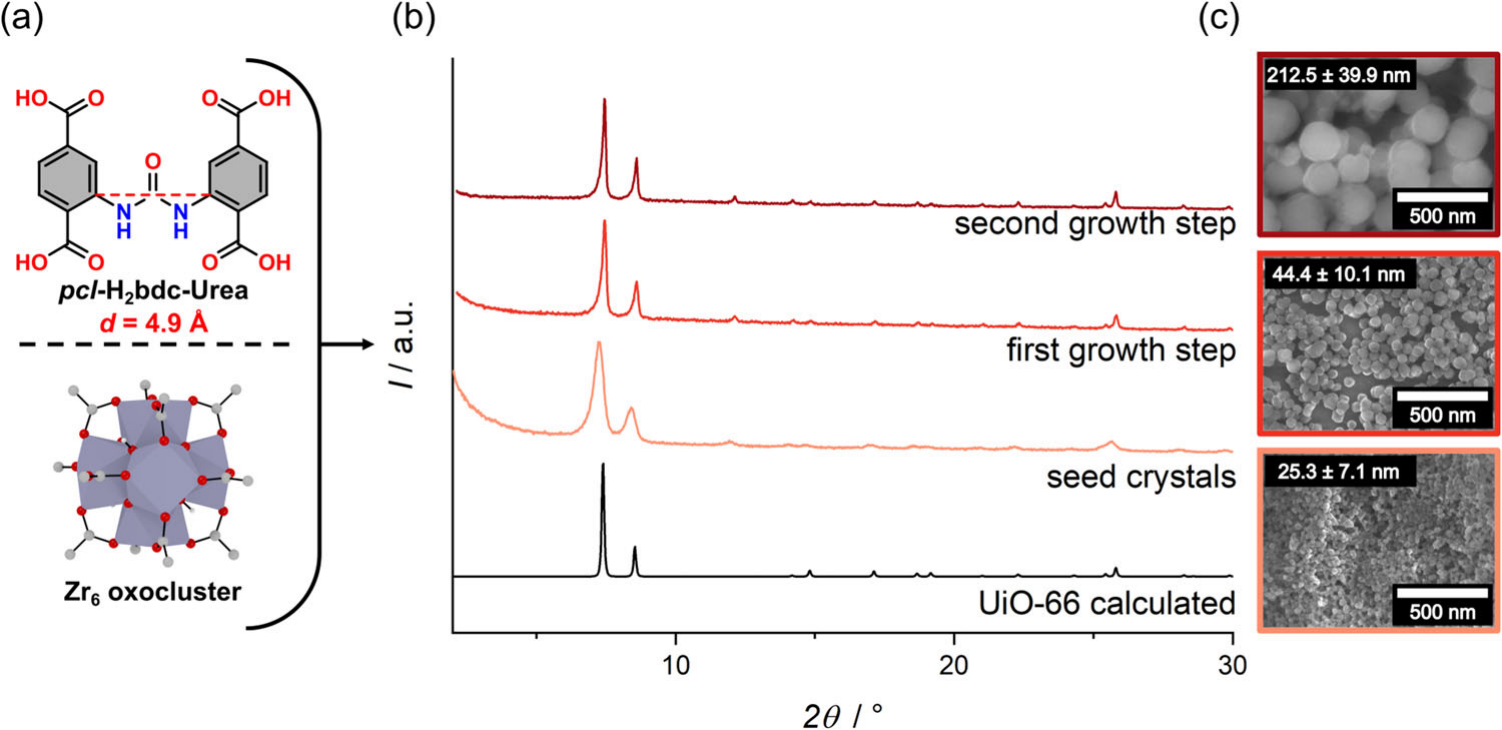
(a) Zr_6_ cluster‐based synthesis of UiO‐66 type framework with pre‐synthetically bridged *pcl*‐H_2_bdc‐Urea linker, (b) powder diffractograms, and (c) corresponding SEM images of *pcl*‐UiO‐66‐Urea. Panels (b) and (c) show particles after initial synthesis (orange), first growth step (red) and second growth step (brown). An increase in crystallinity and particle size is observed with each growth step. A calculated reference diffractogram of UiO‐66 is shown in black.

The resulting diffractograms, SEM images, and particle diameters are shown in Figure 2a.

A decrease in background scattering and in the width of the main reflections is evident with increasing particle size. To confirm the successful introduction of the intact *pcl*‐H_2_bdc‐Urea linker into the framework, ^1^H‐NMR dissolution experiments were carried out, where partial degradation of the *pcl*‐H_2_bdc‐Urea linker to H_2_bdc‐NH_2_ was observed. The ratio of *pcl*‐H_2_bdc‐Urea: H_2_bdc‐NH_2_ increased with increasing particle size from 5.00:1 through 5.56:1 up to 6.25:1, which we attribute to the lower particle surface to bulk ratio. Due to the least amount of linker degradation and possibly the lowest amount of missing cluster defects, all following work was carried out with the largest grown particles. Furthermore, the chemical stability toward various pH ranges and solvents of the framework was confirmed (See Figure S10).

Framework activation via Soxhlet extraction with DCM and subsequent activation at room temperature under dynamic vacuum was required to prevent thermal crosslink degradation (See Figures S6 and S8). Ar physisorption revealed a decrease in porosity with each growth step from 905 m^2^g^−1^ (seed crystals) to 703 m^2^g^−1^ (after two growth steps, Figure S7). The reduction of porosity may be attributed to a higher relative number of intact urea crosslinks and simultaneous defect healing of missing linkers or clusters, which is evident from the decreased uptake in the mesoporous isotherm range of the larger grown particles compared to the initial seed crystals. Therefore, our approach enables dense functionalization without compromising the porosity of the framework.

### 2.3 SerialPED to Prove Trigonal Window Incorporation

#### 2.3.1 UiO‐66

In order to investigate the effect of the urea crosslink on the UiO‐66 framework and confirm our structural considerations, 3D electron diffraction (3DED) measurements at room temperature were first conducted on a regular UiO‐66 sample for comparison. The expected structure was readily determined (see Figure 3c). Based on the anisotropic displacement parameters (ADPs) of the non‐axial carbon atom (site C3), a mean‐square displacement of *λ*_1_ = 0.28(2) Å^2^ in the direction perpendicular to the phenyl ring clearly indicates a vibrational motion (Figure 3d). This sensitivity to such structural subtleties based on electron diffraction data has already been demonstrated in the case of UiO‐67 at room temperature by Samperisi et al. [44]. The *R*_1_(obs) value of the structure refinement converged to be reasonable 20.3% [45, 46].

**FIGURE 3:**
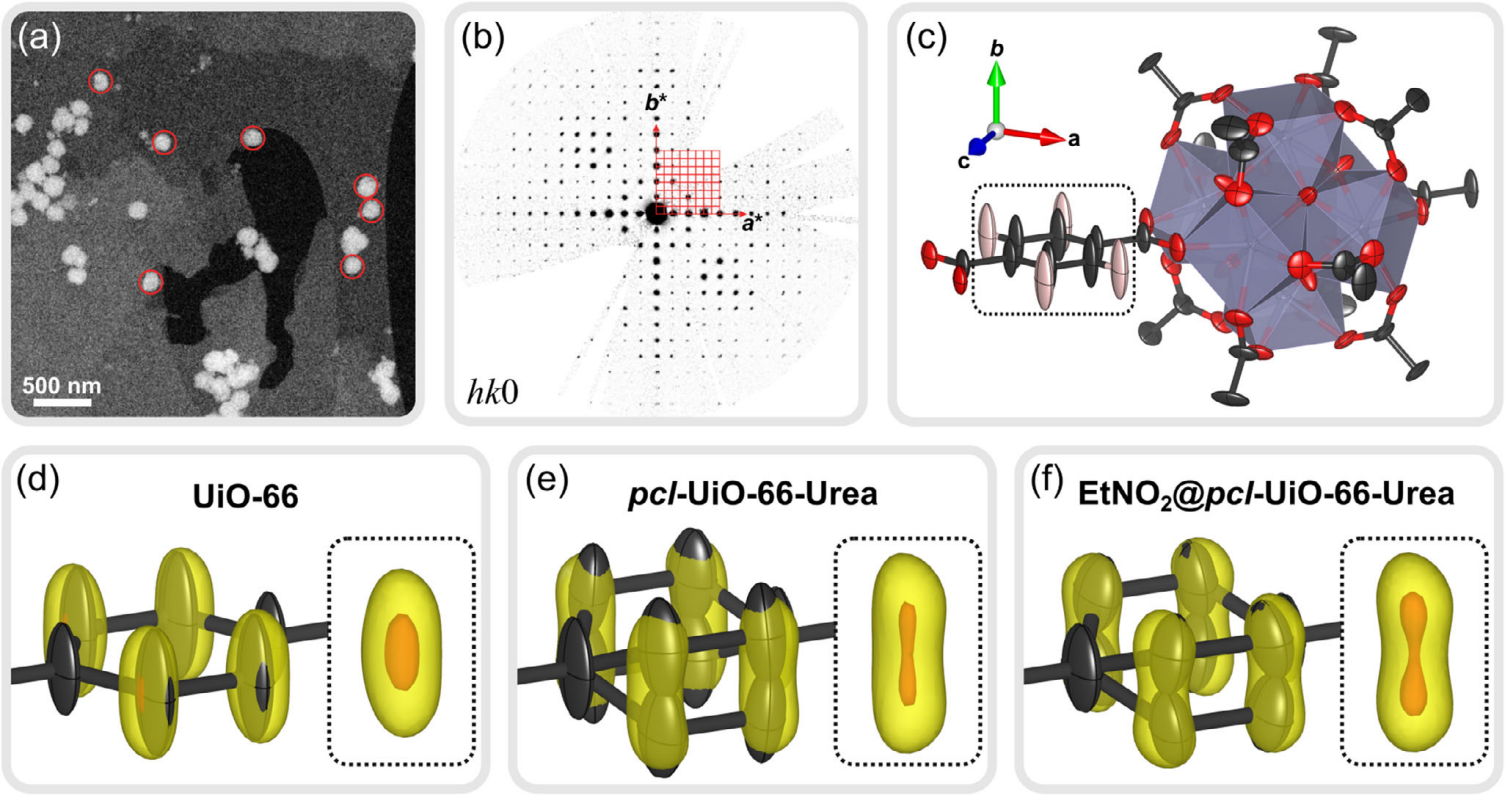
Vibrational motion and split positions obtained by 3DED and SerialPED on UiO‐66 derivates. (a) Exemplary reference image used to select *pcl*‐UiO‐66‐Urea particles. The red circles correspond to the positions where the beam (*d* = 200 nm) was placed to acquire single PED patterns. (b) Exemplary reconstructed crystallographic section *hk*0 from 121 acquired PED patterns from SerialPED data of sample *pcl*‐UiO‐66‐Urea. The reciprocal translation lattice is indicated by the red squares shown. (c) Structure determination of regular UiO‐66 based on 3DED data. Representation of the thermal ellipsoid model (50% probability) of a Zr_6_‐based cluster with one linker. (d‐f) Benzene ring of UiO‐66 structures overlaid with difference Fourier maps Δ*V*(r). Yellow isosurfaces correspond to the 3σ[ΔV(r)] and red isosurfaces to the 5.43σ[ΔV(r)] level. Other atoms are omitted for clarity.

#### 2.3.2 *Pcl*‐UiO‐66‐Urea

In the next step, an attempt was made to also record 3DED data sets of the samples with crosslinking groups, *pcl‐*UiO‐66‐Urea. The particles from the second growth step (Figure 2c), with sizes of about 175–250 nm, are particularly suitable for single‐crystal measurement by 3DED. However, beam sensitivity, rather than particle size, turned out to be the limiting factor. The rapid decomposition of MOFs in the electron beam due to radiolysis and knock‐on damage is a frequently observed phenomenon [46, 47]. Individual particles survived a total electron fluence of less than 0.1 e– Å^−2^, allowing for the acquisition of a maximum of two diffraction patterns per particle before significant beam damage occurred. To circumvent the substantial beam damage, we employed the recently reported SerialPED method [47], combining hundreds of independent precession electron diffraction (PED) patterns from randomly oriented crystals. By using SerialPED, we successfully bypassed the stability constraints that typically limit MOF structural analysis when using electron diffraction. While the initial structural models derived from this method were incomplete (C3 from the benzene rings of the linker were missing), subsequent refinement and inspection of the difference Fourier map revealed a split position for the C3 atom, characterized by an elongated out‐of‐plane density (see Figure 3e). Considering the split position of C3 and further refinement of the structure, it can be seen that the mean displacement of *λ*_1_ = 0.17(2) Å^2^ is reduced by almost 40% compared to regular UiO‐66. This indicates an out‐of‐plane twisting of the linkers to accommodate the slightly shorter urea crosslink. Furthermore, even the nitrogen atom of the crosslinker could be detected in the difference Fourier synthesis.

#### 2.3.3 EtNO_2_@*Pcl*‐UiO‐66‐Urea

The same effect, but stronger and more pronounced λ_1_ = 0.11(1) Å^2^ (Figure 3f), could be observed for the *pcl*‐UiO‐66‐Urea with adsorbed nitroethane (EtNO2@*pcl*‐UiO‐66). The nitrogen atom of the urea linker could also be determined more easily. This could be explained by the filled pores with simultaneous formation of hydrogen bonds to the guest, which should ultimately reduce the vibrational motion of the linker.

The out‐of‐plane twist of the benzene of the linker ring is 23.2(7)°, resulting in a C–C crosslinking distance of 4.77(3) Å (see Figure 1b for comparison). The closest N–N distance in the refined crystal structure is 2.4(2) Å (Figure 4), which is consistent with the expected N–N distance in urea of approximately 2.3 Å. Even a faint signal is visible between the nitrogen atoms in the differential Fourier map, which can be interpreted as an indication of the carbon atom in the urea group (see Figure 4).

**FIGURE 4:**
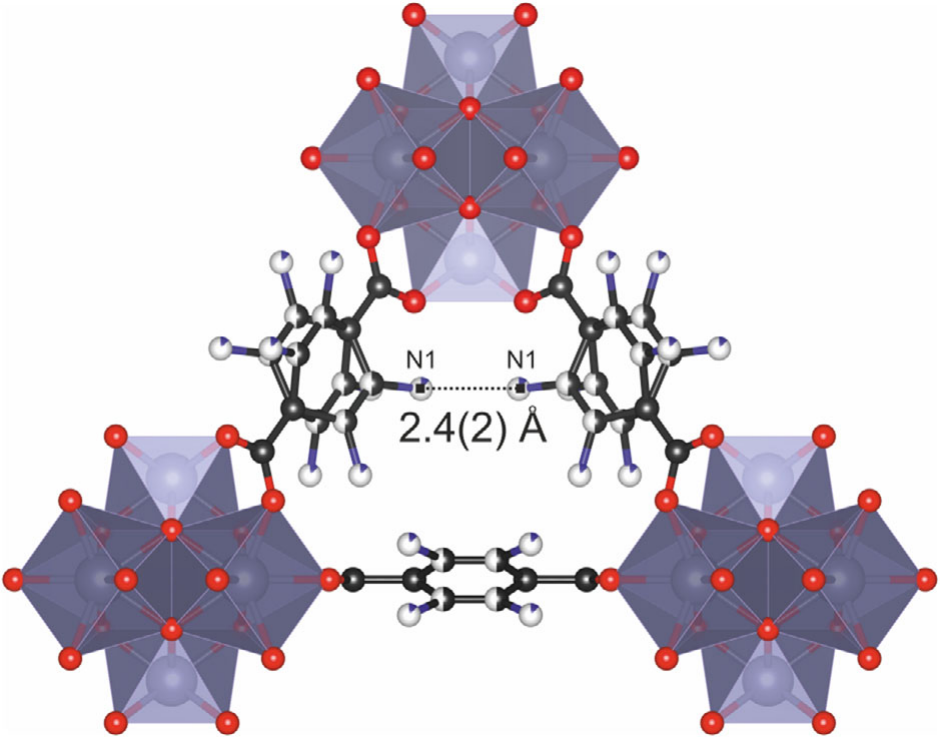
Representation of a trigonal window of the refined crystal structure of EtNO_2_@*pcl*‐UiO‐66‐Urea, viewed along the [110] direction. Partially occupied nitrogen atoms are depicted in blue and carbon atoms in black. Hydrogens have been omitted for clarity.

Thus, based on the SerialPED data, the crystal structure analysis indicates that the incorporation of the cross‐linker was successful. However, deviations in the local chemical environment of the respective trigonal window are to be expected, since the cubic symmetry of the crystal structure cannot fully capture this environment.

### 2.4 Resulting Binding Pocket Structures

Based on the electron crystallography results, a structural model of *pcl*‐UiO‐66‐Urea was developed and refined through force‐field‐based modeling (see Section 2.6, Molecular Dynamics Analysis). It is important to note that no single linker connection pattern was identified because the urea crosslinks show no distinct long‐range ordering within the MOF structure. The MOF is constructed periodically from *pre‐connected* linkers, each dictating the connection pattern of adjacent linkers. This results in a large number of possible connection patterns, which we deem statistically equally probable. Detailed considerations of these structures are provided in the Supporting Information (Section S5.2, Connection Pattern Across Trigonal Pore Windows). For simplicity, only a single boundary structure is considered hereafter.

This boundary structure corresponds to the structure shown in Figure 1b. In this configuration, crosslinks are confined only to the pore windows of four of the eight possible tetrahedral pores. The neighboring four tetrahedral pores consist of non‐blocked trigonal pore faces, as each *pre‐connected* linker is shared between two adjacent tetrahedral pores, but only connects in one direction through the urea bridge. This arrangement results in a structural motif found in approximately one third of all possible connection patterns (See Table S3): The tetrahedral pore with crosslinks confined to its pore windows forms a pocket‐like structure, as the crosslinks block access to three of the four pore windows. The remaining open pore window, resulting from the fact that each tetrahedral pore is spanned by three *pre‐connected* linkers (equivalent to six regular linkers) but possesses four faces, allows guest molecules to enter the pocket. Once inside, guests can interact with the three urea crosslinks. These interactions suggest a strong affinity for guest molecules capable of hydrogen‐bonding to urea groups, such as nitro compounds (Figure 5a). Furthermore, the crosslinks within a single pocket are mutually dependent (when two of the three linkers spanning up a pore window are *pre‐connected*, the third linker has to crosslink across the adjacent pore window). Therefore, all urea groups in the blocked pore windows have a constant distance of 7 Å from one another through the pocket.

**FIGURE 5:**
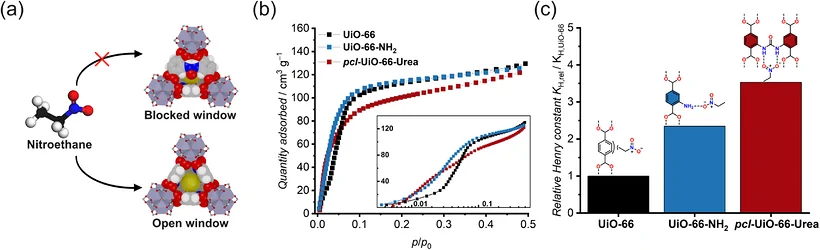
Probing the affinity of formed binding pockets in the structure of pcl‐UiO‐66‐Urea using vapor sorption methods. (a) Representation of a single binding pocket formed by three pre‐connected linkers spanning up a tetrahedral pore. The crosslinks block three of the four pore windows for guest molecules such as nitroethane. The yellow sphere represents pore volume accessible through the remaining open pore window of the pocket structure. (b) Nitroethane vapor sorption isotherms (298 K) and (c) relative Henry constants normalized to UiO‐66. The nitroethane adsorption isotherms in (b) exhibit Type I behavior with pore filling being accomplished at low relative pressures. The inset shows a logarithmic plot of the isotherms, highlighting the different uptake behavior under dilute conditions. The Henry constants shown in (c) correspond to framework affinity towards nitroethane at low pressures. The values are rationalized through the interactions shown above the bars.

### 2.5 Probing Binding Pocket Affinity through Nitroethane Vapor Sorption

To demonstrate the functional versatility of the obtained *pcl*‐UiO‐66‐Urea, we investigated the adsorption of aliphatic nitro compounds at room temperature. As these compounds range from occupational hazards to explosives, their capture and detection are of great societal importance. MOFs have already been successfully employed to address this challenge [48]. Compounds like 2,3‐dimethyl‐2,3‐dinitrobutane (DMDNB), which feature two nitro groups, are particularly noteworthy (see binding energy calculation in Table S4). The urea groups within the identified pockets in *pcl*‐UiO‐66‐Urea are positioned at a precise distance from one another, potentially enabling simultaneous hydrogen bonding to both nitro groups, resulting in very strong interactions. While future experimental studies will target such compounds, this work focuses on the vapor adsorption of nitroethane. Nitroethane is chemically similar to DMDNB, but is more compatible with standard sorption instruments because it is a liquid at room temperature. Non‐functionalized UiO‐66 and an amino‐functionalized derivative (UiO‐66‐NH_2_) were used as references. Their syntheses and characterization are described in the Supporting Information (Section S3.2, Synthesis of UiO‐66 and UiO‐66‐NH_2_).

The resulting nitroethane sorption isotherms are shown in Figure 5b and show type‐I behavior [49]. For all UiO‐66 derivatives, micropore filling is completed before *p*/*p*_0_ = 0.1. A bend in the low‐pressure region of UiO‐66 is notable, which we attribute to the filling of the tetrahedral pores. In contrast, for the modified derivatives, the interaction is dominated by the functional groups, resulting in continuous filling. Affinity at low nitroethane concentrations was quantified using the Henry constant, which is a common affinity metric in vapor adsorption and is determined from the slope of the linear region of the adsorption isotherms at low relative pressures (Figure S12) [50, 51]. The values in Figure 5c were normalized to the mean Henry constant of UiO‐66 to enable comparison.

It is evident that while UiO‐66‐NH_2_ shows an intermediate increase of roughly 2.3‐fold in affinity compared to UiO‐66, *pcl*‐UiO‐66‐Urea displays an approximately 3.5‐fold increase in affinity. This enhancement is attributed to the bifurcated hydrogen‐bonding interaction between the urea and nitro groups in *pcl*‐UiO‐66‐Urea, as shown in Figure 5c, whereas UiO‐66 und UiO‐66‐NH_2_ exhibit only single or no hydrogen bonding, respectively.

### 2.6 Molecular Dynamics Analysis

Computational simulations were performed to validate the effectiveness of the formed binding pockets in capturing nitroethane through hydrogen bonding interactions. For this, the trajectories of single molecules were simulated using force‐field molecular dynamics (MD) calculations.

The force field calculations were performed using the Forcite module of the Materials Studio 23.1.1.7 software package developed by BIOVIA [52]. The simulations were carried out using a force field based on Dreiding [53] supplemented by zirconium parameters from the UFF4MOF [54]. The starting structure for the *pcl*‐UiO‐66‐Urea was created manually by connecting linkers in a UiO‐66 structure. The charges were calculated using an iterative process. For this, charges are calculated using the charge equilibration method (QEq) as implemented in Materials Studio. The resulting structure with partial charges is then optimized with a geometry optimization run. Afterwards, new charges are calculated using QEq again. This process is repeated, alternating charge calculation and optimization until all individual partial charges differ less than 1e‐5 e between iterations.

Two probe molecules were selected to test the effectiveness of the designed MOF. As previously described, nitroethane was chosen to demonstrate the affinity of *pcl*‐UiO‐66‐Urea towards nitro groups, supplementing the vapor sorption results. Propane was used for comparison as it is similar in size to nitroethane, but lacks hydrogen bonding interactions with the framework. Each molecule was placed separately within the MOF at various starting positions. In some instances, molecules were placed directly inside a functionalized tetrahedral pore. To test the mobility toward these pockets, the molecules were also placed within unfunctionalized tetrahedral pores and the octahedral pore. Molecular dynamics simulations were then carried out (*NVT*, 300 K, 50 ns), and the trajectories of the molecules were tracked by recording the coordinates of the nitrogen atom in nitroethane and the coordinates of one of the terminal carbon atoms in propane, respectively.

The results are presented as simplified 3D models shown in Figure 6, showing three distinct snapshots from the beginning, middle, and end of the simulation. Additional trajectories with different starting positions are provided in the Supporting Information (Section S6.1 Additional trajectory snapshots). As shown in Figure 6a, the nitroethane molecule starts inside an unfunctionalized tetrahedral pore. The molecule then diffuses throughout the pore system until it enters a functionalized pocket. From that point onwards, the nitroethane molecule is confined to that pocket. It can reorient, but cannot exit the pocket. In contrast, propane diffusion does not cease upon entering or leaving functionalized pockets (Figure 6b). This effect is consistent regardless of the starting position, as further detailed in the Supporting Information. These results suggest that MOFs featuring such functionalized pockets show a very high affinity for nitro compounds.

**FIGURE 6:**
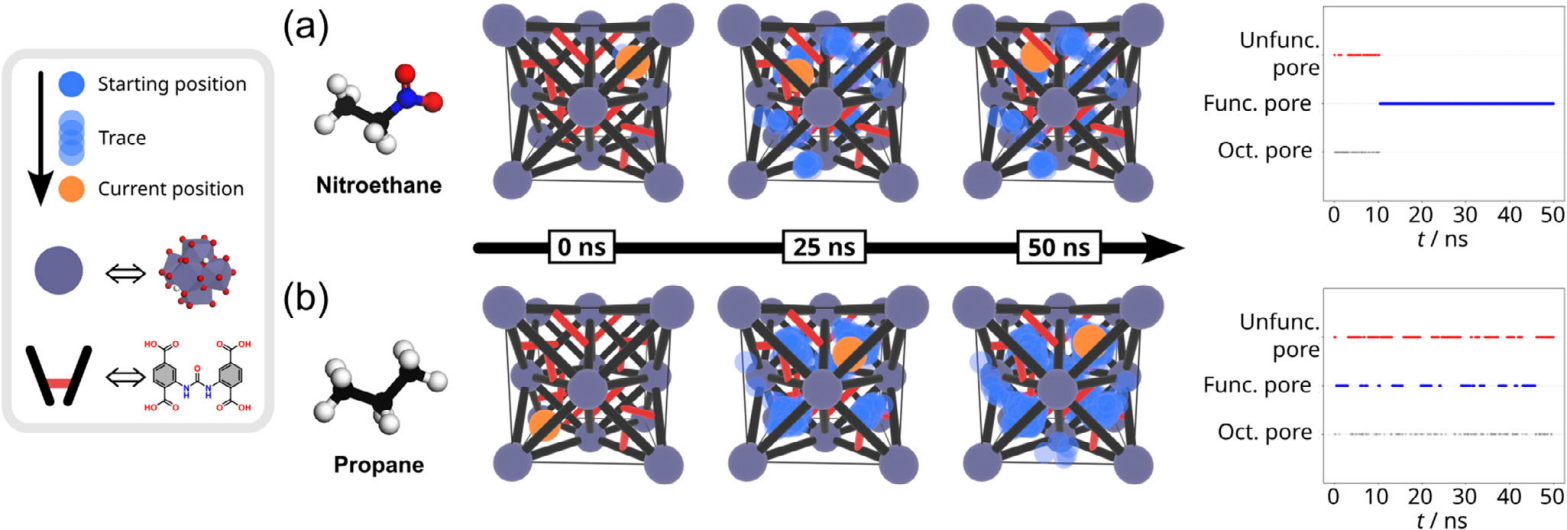
Molecular dynamics simulations of single (a) nitroethane and (b) propane molecules in *pcl*‐UiO‐66‐Urea, respectively. Trajectories are shown for three time points (start, middle, and end) and visualized using colored spheres. An orange sphere highlights the current position of the molecule, a solid blue sphere represents the starting position, and all positions between those points are shown as faded blue spheres. For clarity, the Zr‐clusters are represented by purple spheres and the linkers by black rods. To highlight the connection pattern, the bridging urea groups are represented by red rods. This model neglects potential rotation and movement of the linkers, but enables easy visualization of the trajectory of the probe molecules. Additionally, the graphs to the right indicate the type of pore occupied by the molecule during the simulation.

In addition to *pcl*‐UiO‐66‐Urea, UiO‐66‐NH_2_ was chosen as a reference material equivalent to the experimental sorption experiments. As can be seen in the Supporting Information (Figure S25), nitroethane in UiO‐66‐NH_2_ behaves similarly to nitroethane in *pcl*‐UiO‐66‐Urea. To supplement these qualitative analyses of the trajectory, mean square displacements (MSDs) were calculated.

In Table 1, the MSDs of the tracked atoms between every recorded snapshot (50 ps) are shown. The results are averaged across 10 independent simulations. Nitroethane has a lower MSD in *pcl*‐UiO‐66‐Urea than in UiO‐66‐NH_2_ due to stronger interaction with the MOF, leading to less frequent reorientation within the pore and less movement during these reorientations. In contrast, the reverse is observed for propane in the two MOFs. Here, since no strong interactions with the urea groups are present, the steric constraints of the pores containing crosslinked linkers result in a significant increase in MSD connected with the propane molecule changing between pore types frequently (as shown in Figure 6). The increased MSD of nitroethane in UiO‐66‐NH2 when compared to propane is a result of the methodology used to calculate these MSDs. As explained above, single atoms (nitrogen and terminal carbon) were tracked. Nitroethane frequently reorients between functional groups and IBUs to form hydrogen bonds. The interactions of propane with the framework are not directional, resulting in less reorientation and a lower MSD.

**TABLE 1:** Mean square displacement < *x* ^2^> of nitroethane and propane in UiO‐66‐NH_2_ and *pcl*‐UiO‐66‐Urea, respectively.

Molecule	MOF	Starting point	<*x* ^2^>/Å ^2^
Nitroethane	UiO‐66‐NH_2_	tet. pore	10.5 ± 0.3
		oct. pore	10.2 ± 0.7
	*pcl*‐UiO‐66‐Urea	func. pore	1.0 ± 1.2
		unfunc. pore	4.6 ± 4.6
		oct. pore	5.4 ± 3.5
Propane	UiO‐66‐NH_2_	tet. pore	8.0 ± 1.1
		oct. pore	7.8 ± 1.4
	*pcl*‐UiO‐66‐Urea	func. pore	12.4 ± 4.1
		unfunc. pore	14.8 ± 5.7
		oct. pore	14.0 ± 3.4

*Note*: The displacements are calculated every 50 ps and differentiated by the starting position of the molecule. The results are averaged over 10 individual simulations.

## 3 Conclusion

In this work, we have demonstrated a strategy to introduce local asymmetry into the highly symmetric UiO‐66 framework to create well‐defined chemical environments. Through straightforward structural considerations, urea groups were placed with precise distances and positioning within the structure. This was achieved by pre‐synthetically bridging two linkers with a urea moiety, which restricts the crosslink to the tetrahedral pore windows due to its constrained length. The specific arrangement of these crosslinks was unambiguously confirmed using electron diffraction methods, while the enhanced functionality of the resulting pockets was demonstrated through nitroethane vapor sorption and validated through modelling studies. This concept is applicable to any MOF system provided that a *pre‐connected linker* can be synthesized to bridge the shortest linker–linker distance in the framework. Thus, it eliminates the need to design a MOF from scratch for an application where precise binding site design, such as catalysis or selective adsorption, is required, as existing structures can be modified using our presented strategy. Therefore, through the use of *pre‐connected linkers*, our method moves beyond simple functionalization of linkers toward a general strategy for designing pores in MOFs suitable for molecular recognition.

## Author Contributions

**Tobias Hennig**: writing – original draft, investigation, formal analysis, writing – review and editing, data curation, visualization, methodology. **Erik Rohloff**: software, writing – original draft, writing – review and editing, formal analysis, investigation, data curation, visualization. **Verena Drewes**: investigation, formal analysis. **Adrian Hannebauer**: investigation, formal analysis. **Sergi Plana‐Ruiz**: investigation, formal analysis, software, methodology, writing – review and editing. **Yaşar Krysiak**: investigation, formal analysis, software, visualization, writing – original draft, writing – review and editing. **Andreas M. Schneider**: writing – review and editing, supervision, project administration, funding acquisition. **Andreas Schaate**: resources, writing – review and editing, project administration, conceptualization, supervision, funding acquisition.

## Conflicts of Interest

The authors declare no conflicts of interest.

## Supporting information




**Supporting File**: anie74752‐sup‐0001‐SuppMat.docx.

## Data Availability

The data that support the findings of this study are available in the Supporting Information of this article.
