# Supplementary material for: Formation of Highly Selective Binding Pockets in UiO‐Type MOFs From Bridged Linkers
Source: Angew Chem Int Ed Engl. 2026 Sep 10;65(44):e5435518. doi: 10.1002/anie.5435518 (PMC13703899; doi:10.1002/anie.5435518)
Supplement: Supplementary file 1 [file ANIE-65-e5435518-s001.docx]

# Supporting Information: Formation of highly selective binding pockets in UiO-type MOFs from bridged linkers

Tobias Hennig,^[a]^ Erik Rohloff,^[a]^ Verena Drewes,^[a]^ Adrian Hannebauer,^[a]^ Sergi Plana-Ruiz,^[c]^ Yaşar Krysiak,^[a,d]^ Andreas M. Schneider,^[a,b]^ Andreas Schaate^*[a,b]^

[a]	T. Hennig, E. Rohloff, V. Drewes, Dr. A. Hannebauer, Dr. Y. Krysiak, Dr. A. M. Schneider, Dr. A. Schaate
Institute of Inorganic Chemistry
Leibniz University Hannover
Callinstr. 9, 30167 Hannover, Germany
E-mail: andreas.schaate@acb.uni-hannover.de

[b]	Dr. A. M. Schneider, Dr. A. Schaate
Cluster of Excellence PhoenixD (Photonics, Optics and Engineering – Innovation Across Disciplines)
Leibniz University Hannover
Welfengarten 1A, 30167 Hannover, Germany

[c]	Dr. S. Plana-Ruiz
Crystallography and Mineralogy Unit, Department of Geology
Universitat Autònoma de Barcelona
Campus de Bellaterra, Edifici B, Carrer de la Fortuna, 08193 Bellaterra, Spain

[d]	Dr. Y. Krysiak
Laboratory for Nano and Quantum Engineering
Leibniz University Hannover
Schneiderberg 39, 30167 Hannover, Germany

Content

Supporting Information: Formation of highly selective binding pockets in UiO-type MOFs from bridged linkers	1

S1. Materials and methods	3

S1.1 Materials	3

S1.2 Methods	3

S2. Linker synthesis	5

S3. MOF synthesis	6

S3.1. Synthesis of *pcl*-UiO-66 Urea	6

S3.2 Synthesis of UiO-66 and UiO-66-NH_2_	6

S4. Additional characterization	7

S4.1. Linker characterization	7

S4.2. *pcl*-UiO-66-Urea particle growth and stability	10

S4.3. Electron crystallography	13

S4.4. Vapor sorption and reference MOF characterization	15

S5. Structural considerations	17

S5.1 Connection types in the UiO-66 structure	17

S5.2 Connection pattern across trigonal pore windows	18

S6. Additional simulation data	19

S6.1 Additional trajectory snapshots	19

S6.2 Additional pore mobility analysis	21

S6.3 Binding energy of DMDNB and nitroethane	22

References	23

## S1. Materials and methods

### S1.1 Materials

Chemicals were purchased from commercial vendors and used as received without further purification, unless otherwise stated. 1,1’-Carbonyldiimidazole (CDI, Sigma-Aldrich, 97 %), 2-Aminoterephthalic acid (H_2_bdc-NH_2_, Alfa Aesar, 99 %), Acetic acid (Sigma-Aldrich, 99.7 %), Acetone (Carl Roth, 99.5 %), Chloroform-d (CDCl_3_, Deutero, 99.8 %), Caesium fluoride (CsF, Sigma-Aldrich, 99 %), Deuterium chloride 37% in D_2_O (DCl in D_2_O, Deutero, 99.5 %), Dichloromethane (DCM, Carl Roth, 99.5 %), Dimethylsulfoxide-d_6_ (DMSO-d_6_, Deutero, 99.8 %), Ethanol (EtOH, Carl Roth, 96 %), Nitroethane (EtNO_2_, S3 Chemicals, 99.5 %), Hydrogen chloride 37% in H_2_O (HCl, Roth, technical), Isopropanol (Carl Roth, 99.5 %), Methanol (MeOH, Carl Roth, 99 %), N,N’-Dimethylformamide (DMF, Sigma-Aldrich, 99.8 %), Potassium trimethylsilanoate (TMS-OK, BLDPharm, 96%), Terephthalic acid (H_2_bdc, Sigma-Aldrich, 98 %), Zirconium(IV) acetylacetonate (Zr(acac)_4_, Sigma-Aldrich, 99.5 %) and Zirconium(IV) chloride (ZrCl_4_, Sigma-Aldrich, 99.5 %) were used as received. Dry Tetrahydrofuran (THF, Sigma-Aldrich, 99.9 %) was obtained from a glovebox equipped with a solvent purification system. Dimethyl 2-aminoterephtalate (Sigma-Aldrich, 97 %) was recrystallized from H_2_O/EtOH prior to use to remove impurities.

### S1.2 Methods

**Powder X-Ray diffraction (PXRD)** was carried out in transmission mode using a Stoe Stadi-P diffractometer operated with Ge(111)-monochromatized Cu-K_α_ radiation (λ =1.54060 Å). A Mythen 1K detector was used and the diffractograms were recorded from 2 to 50° 2Θ.

**Argon physisorption** measurements were performed at 87 K on a 3Flex instrument from micromeritics. The solvent-exchanged MOF powder samples (30 mg) were activated for 20 h before each measurement, as previously described. Data analysis was performed using the associated 3Flex V6.03 software. BET surface areas were determined by the BET-auto function of the software.

**Static vapor sorption** measurements for nitroethane were performed on a 3Flex instrument from micromeritics and on a vapor 100C from 3P instruments. For this, commercial nitroethane (99.5%) was stored over molecular sieves to remove water. The vapor source was degassed through three freeze-pump-thaw-cycles prior to measurement. Sample tube temperatures were maintained at 25°C using a water bath. Solvent-exchanged MOF powder samples (30 mg) were activated for 20 h before each measurement, as previously described.

**H-NMR and proton-decoupled ^13^C-NMR** spectra of linkers were recorded on a JEOL JNM-ECZS Spectrometer at 400 MHz and 100 MHz, respectively. Spectra were processed using the MestreNova software and referenced to the solvent signal as an internal standard.

MOF samples were analyzed via liquid ^1^H-NMR spectroscopy using a digestion procedure reported by ROY et al.^[1]^ For this, 10 mg of MOF and 10 mg CsF were added to 0.7 mL DMSO-d6. Subsequently, five drops of 37 % DCl in D_2_O were added and the suspension was sonicated at 60°C for 1 h. Then, 30 mg of K_2_CO_3_ was added and the suspension was agitated for 5 min before centrifugation. The clear supernatant was then transferred to an NMR tube and measured quantitatively with a relaxation delay of 100s.

**Three-dimensional Electron Diffraction (3DED)** measurements of regular UiO-66 were carried out with a transmission electron microscope (TEM) FEI Tecnai F30 S-TWIN equipped with a field emission gun (300 kV) The powdered samples were dispersed in ethanol and deposited onto a carbon-coated copper grid. A condenser aperture of 10 μm and mild illumination settings (gun lens 8, spot size 8) were used to produce a semi-parallel beam of 200 nm in diameter (0.212 e-/Å^2^s). Imaging and diffraction were performed in scanning transmission electron microscopy (STEM) mode whereby images were collected by a FISCHIONE high-angular annular dark field (HAADF) detector. Diffraction patterns were collected continuously by an ASI Timepix1 detector (512 × 512 pixels) at 300 mm Camera Length (CL) and exposure time of 0.5 s. The continuous-rotation 3DED data were collected using the acquisition module PyFast-ADT.^[2]^

**Serial electron diffraction** was performed on powdered samples (*pcl*-UiO-66-Urea, EtNO_2_@*pcl*-UiO-66-Urea) dispersed in ethanol using an ultrasonic bath and deposited onto a carbon-coated copper grid. The acquisition of the precession-integrated SerialED (SerialPED) data was performed with a JEOL F200 equipped with a cold field emission electron gun operated at 200 kV (0.02508 Å) in STEM mode (probe size 8, 10 µm condenser aperture) and a post-column Gatan OneView camera (16-bit CMOS-based and optical fibre-coupled detector of 4096 × 4096 pixels, 15 mm physical pixel size). A quasi-parallel beam in STEM was manually aligned,^[3]^ so that the most parallel condition (0.09 x 0.02 mrad of convergence semi-angle at FWHM) could be attained with a beam diameter of 200 nm (FWHM) with an electron flux density ranging from 36 to 51 e^–^ Å^-2^ s^-1^ for the diffraction pattern acquisition (exposure time of 0.25 s, electron fluence ranging from 9-13 e^–^ Å^-2^). Small convergence semi-angles are desired for ED patterns in order to obtain statistically significant counts for the weakest reflections. A more convergent electron probe was used for the STEM reference images acquired with a JEOL high-angle annular dark-field (HAADF) detector. Gatan Digital Micrograph script were used for ease of use (freely available at https://github.com/sergiPlana/TEMEDtools/tree/main/STEMSerialED).^[4]^ The precession of the electron beam at 100 Hz frequency was enabled by a P2000 prototype precession unit provided by NanoMEGAS SPRL. For data reduction of the *pcl*-UiO-66-urea and EtNO_2_@*pcl*-UiO-66-Urea samples, 121 of 171 and 172 of 207 recorded raw images were used to extract reflection intensities for kinematical and dynamical refinement. Data reduction were performed with PETS2 (Version 2.3.20260217.0937).^[5]^ For indexing, a template-matching algorithm^[6]^ implemented in PETS2 that assigns orientation angles with respect to a reference orientation matrix for each pattern was used. Structure solution, difference Fourier mapping and least-squares (dynamical) refinement were performed with the JANA2020 software (Version 2.2).^[7]^ Thus, the independent PED patterns were successfully indexed by template-matching based on the respective lattice parameters derived from Pawley fit^[8]^ (*F m*-3*m*, a = 20.794 Å or 20.775 Å) performed in DASH^[9]^. By knowing the relative crystallographic orientations of the individual PED patterns the experimental crystallographic sections could be reconstructed (Figure 3a) and the Laue group of the UiO-66 framework confirmed (*m*-3*m*, *R*_int_(all) = 11.8 % or *R*_int_(all) = 11.4 %). The structures could be solved with Superflip^[10]^ in space group *F m*-3*m*. One missing carbon atom (C3) was placed on the out-of-plane position (Wyckoff position 192l) and the structure refined with a relative occupancy fixed to 0.5 for C3. The *wR*(all) value drops down significantly to 20.5 % or 21.9 % and the ADPs of C3 converges to a relative moderate amplitude in the plane direction of linkers benzene ring. Furthermore, even the nitrogen atom of the crosslinker could be detected in the difference Fourier synthesis. Consequently, the *wR*(all) value converged to 19.7 % and 21.5 % respectively.

**Electrospray ionization mass spectrometry (ESI-MS)** was performed using a Waters QTof Premier instrument at the Centre for Mass Spectrometry at Leibniz University Hannover.

**Scanning electron microscope (SEM)** images were captured using a Regulus SU8200 (Hitachi). Images were typically recorded at a voltage of 10 kV and a current of 10 µA.

**Attenuated total reflectance Fourier-transform infrared (ATR-FTIR)** spectroscopy was performed using a Bruker Tensor 27 spectrometer. The absorbance was recorded within the range of 4000 to 600 cm⁻¹ with a resolution of 1 cm⁻¹. Powder samples were directly placed onto the ATR crystal for analysis.

**Thermogravimetric analysis - coupled mass spectrometry (TGA-MS)** was performed on a Netzsch STA 409 PC Luxx thermobalance coupled to a Netzsch QMS 403 D Aëolos mass spectrometer. A heating rate of 5 K/min with a flow rate of 30 ml min^-1^ O_2_ / 70 ml min^-1^ N_2_ was employed.

**Ultraviolet/Visible (UV/vis) spectroscopy** of powder samples was recorded with a Cary 4000 (Agilent Technologies) in diffuse reflectance in the spectral range from 250 to 600 nm using the software “Scan”. A praying mantis sample holder attachment was employed.

## S2. Linker synthesis

Linker synthesis steps were performed under inert gas (Ar) using standard SCHLENK technique in flame-dried glassware charged with magnetic stir bars.

**Scheme S 1.** Synthesis of *pcl*-H_2_bdc-Urea linker **2.**

**Experimental Procedures of 1-2**

**Preparation of *pcl*-(OMe)_2_bdc-Urea (1):** This compound was prepared according to a modified literature procedure.^[11]^ To a two-neck 100 mL SCHLENK flask affixed to a reflux condenser was added THF (25 mL). Subsequently, dimethyl 2-aminoterephtalate (2.092 g, 10.0 mmol, 1.00 eq) was added and stirred until dissolved, followed by the addition of CDI (0.972 g, 6.0 mmol, 0.60 eq). The reaction mixture was heated to reflux and additional CDI (0.300 g, 1.8 mmol, 0.18 eq) was added after 2, 4, 6, 8, 24, 26, 28 and 30 h. The precipitated product was filtered off, thoroughly washed with THF and dried under vacuum to yield 1.406 g (63 %) of **1** as a white, fluffy product.

Analytical data for **1**: ^1^H NMR (400 MHz, CDCl_3_) *δ* 10.79 (s, 2H), 9.20 (d, J = 1.7 Hz, 2H), 8.10 (d, J = 8.4 Hz, 2H), 7.69 (dd, J = 8.4 Hz, 1.7 Hz, 2H), 3.97 (d, 12H); ^13^C NMR (100 MHz, CDCl_3_) *δ* 168.6, 166.4, 151.9, 142.6, 135.5, 131.1, 122.3, 120.9, 117.7, 52.9, 52.7; ESI-MS: Exact mass calculated for C_21_H_20_N_2_O_9_Na [M+Na]^+^, 467.10. Found: 467.10. The ^1^H NMR spectrum is in good agreement with reported values.^[12]^

**Preparation of *pcl*-H_2_bdc-Urea (2):** This compound was prepared according to a modified literature procedure.^[11]^ To a 100 mL pear flask was added *pcl*-(OMe)_2_bdc-Urea (**1**) (1.406 g, 3.2 mmol, 1.00 eq), TMS-OK (4.058 g, 32.0 mmol, 10.00 eq) and THF (50 mL). The flask was affixed to a reflux condenser and the mixture was stirred vigorously under reflux overnight. Afterwards, the solvent was removed under reduced pressure and the residue was dissolved in water. The solution was cooled in an ice bath and acidified to *p*H 1 using 1 M HCl, which induced the formation of a yellow precipitate. The product was filtered off, washed thoroughly with water and then with MeOH and dried under vacuum again to yield 1.137 g (93 %) of **2** as a yellow solid.

Analytical data for **2**: ^1^H NMR (400 MHz, DMSO-d_6_) *δ* 13.28 (b, 4H), *δ* 10.72 (s, 2H), 8.84 (d, J = 1.7 Hz, 2H), 8.02 (d, J = 8.2 Hz, 2H), 7.62 (dd, J = 8.2 Hz, 1.7 Hz, 2H); ^13^C NMR (100 MHz, CDCl_3_) *δ* 169.0, 166.7, 151.7, 141.4, 134.5, 131.0, 121.5, 121.4, 120.2; ESI-MS: Exact mass calculated for C_17_H_11_N_2_O_9_ [M-H]^-^, 387.04. Found: 387.04.

## S3. MOF synthesis

### S3.1. Synthesis of *pcl*-UiO-66 Urea

**Precipitation of Zr_6_-Oxoclusters:** Zr_6_-Oxoclusters with the mean sum formula Zr_6_O_4_(OH)_4_(C_2_H_3_O_2_)_8_(H_2_O)Cl_3_ were synthesized according to the procedure reported by Serre *et al.^[13]^* For this, ZrCl_4_ (100 g, 0.43 mol) was heated in a mixture of acetic acid (150 mL) and isopropanol (250 mL) under stirring at 120 °C for 1 h. The precipitated Zr_6_-Oxoclusters were isolated through filtration, washed thoroughly with acetone and dried under vacuum.

**Seed particles:** To a 9 mL reaction vial with a stir bar, Zr_6_-Oxoclusters (45.0 mg, 0.04 mmol, 0.16 eq, corresponding to 1 eq of "Zr"), water (0.943 mL, 52.16 mmol, 250 eq) and acetic acid (0.430 mL, 7.51 mmol, 36 eq) were added. The mixture was stirred until clear, after which DMF (0.808 mL, 10.43 mmol, 50 eq) was added. Lastly, *pcl*-H_2_bdc-Urea (40.5 mg, 0.10 mmol, 0.5 eq) was added and the resulting suspension was stirred for 7 d at room temperature. Afterwards, the precipitate was isolated by centrifugation, washed once with DMF and twice with EtOH, before it was dried under vacuum. For activation, samples were subjected to Soxhlet extraction with DCM for 24 h, followed by guest removal under dynamic vacuum for 20 h without heating.

***pcl*-UiO-66 Urea:** The synthetic procedure used for the seed particles was followed. Before adding the linker, 5 mg of *pcl*-UiO-66-Urea seed particles were added to the reaction tube and sonicated until homogeneously dispersed. Afterwards, *pcl*-H_2_bdc-Urea (40.5 mg, 0.10 mmol, 0.5 eq) was added and the reaction mixture was treated as previously described. For activation, samples were subjected to Soxhlet extraction with DCM for 24 h, followed by guest removal under dynamic vacuum for 20 h without heating.

### S3.2 Synthesis of UiO-66 and UiO-66-NH_2_

**UiO-66:** To a teflon-capped 100 mL pyrex glass vessel was added DMF (25.0 mL, 325.00 mmol, 500 eq), ZrCl_4_ (150.5 mg, 0.65 mmol, 1 eq), water (0.047 mL, 2.60 mmol, 4 eq) and acetic acid (0.370 mL, 6.50 mmol, 10 eq). The mixture was sonicated until a clear solution was obtained, after which H_2_bdc (107.3 mg, 0.65 mmol, 1 eq) was added. The suspension was again sonicated until clear, after which the vessel was sealed and heated at 120 °C for 24 h. Afterwards, the precipitate was isolated through centrifugation and washed once with DMF and twice with EtOH, before it was dried under vacuum. For activation, samples were subjected to Soxhlet extraction with EtOH for 24 h, followed by being heated at 120 °C for 20 h under dynamic vacuum to remove guest molecules. Characterization data are provided in Figure S 13, Figure S 14, Figure S 15 and Figure S 16.

**UiO-66-NH_2_:** In order to prevent linker formylation^[14]^ and formation of -NH_3_^+^Cl moieties within the resulting framework,^[15]^ a DMF-, formic acid- and chlorine-free synthesis by AVCI-CAMUR *et al.* was adapted.^[16]^ For this, Zr(acac)_4_ (389.7 mg, 0.80 mmol, 1 eq) and H_2_bdc-NH_2_ (145.0 mg, 0.80 mmol, 1 eq) were dispersed in water (1.0 mL, 55.20 mmol, 69 eq) and acetic acid (1.0 mL, 17.60 mmol, 22 eq) in a 9 mL reaction vial with a stir bar. The mixture was stirred at room temperature for 72 h. Afterwards, the precipitate was isolated through centrifugation and washed once with water and thrice with EtOH, before it was dried under vacuum. For activation, samples were subjected to Soxhlet extraction with EtOH for 24 h, followed by heated at 120 °C for 20 h under dynamic vacuum to remove guest molecules. Characterization data are provided in Figure S 13, Figure S 14, Figure S 15 and Figure S 16.

## S4. Additional characterization

### S4.1. Linker characterization

**Figure S 1.** ^1^H-NMR spectrum of *pcl*-(OMe)_2_bdc-Urea **1** in CDCl_3_.

**Figure S 2.** ^13^C-NMR spectrum of *pcl*-(OMe)_2_bdc-Urea **1** in CDCl_3_.

**Figure S 3.** ^1^H-NMR spectrum of *pcl*-H_2_bdc-Urea **2** in DMSO-d6.

**Figure S 4.** ^13^C-NMR spectrum of *pcl*-H_2_bdc-Urea **2** in DMSO-d6. Signal of residual methanol from washing is denoted with a dot symbol.

**Figure S 5.** ^1^H-NMR spectrum of the benzoyleneurea decomposition product of *pcl*-H_2_bdc-Urea in DMSO-d6.

### S4.2. *pcl*-UiO-66-Urea particle growth and stability

The thermal and chemical stability of *pcl*-UiO-66-Urea is further examined in the following. The urea crosslinks are thermally labile and start decomposing just above room temperature (Figure S 6) through the release of CO_2_. The framework crystallinity is unaffected by the crosslink degradation, as the diffractograms indicate crystalline materials regardless of decomposition degrees of the original linker. Furthermore, it was expected that the *de novo* synthesized *pcl*-UiO-66-Urea seed particles contain high defect densities as a result of the employed room temperature synthesis method, which was originally developed to obtain highly defective nanoparticles. To probe the sorptive properties of the intact functionalized framework, we expanded the synthesis strategy with two additional growth steps. These steps resulted in a measurable reduction in mesoporosity associated with defects in UiO-66 (Figure S 7) and an increase in the ratio of intact *pcl*-H_2_bdc-Urea linker to its decomposition product H_2_bdc-NH_2_ (Figure S 8, Figure S 9). Furthermore, this growth procedure was essential to produce particles of sufficient size and crystallinity for electron diffraction measurements.

Due to the thermal lability of the urea crosslinks, sample handling and processing were strictly maintained at or near room temperature. We observed significant linker decomposition when activating the framework through Soxhlet extraction with higher boiling point solvents such as acetone (56 °C) or ethanol (78 °C). To preserve the crosslinks, we implemented a gentle activation protocol using DCM (40 °C) for the Soxhlet extraction. Subsequent solvent removal was performed under dynamic vacuum without heat treatment to ensure the urea crosslinks stay intact for sorption experiments (Figure S 16).

Nonetheless, *pcl*-UiO-66-Urea still showed the high chemical stability of its UiO-66 parent. In order to investigate the shelf stability, samples were stored for several months under ambient conditions exposed to atmosphere. Afterwards, stored samples were submerged in various solvents and under various aqueous conditions to investigate chemical stability after prolonged storage periods (Figure S 10). Crystallinity was retained in all cases (except for pH 13 treatment, under which regular UiO-66 also degrades). The linker composition remained unchanged between the stored samples before or after solvent submersion under any conditions. However, due to the prolonged storage period, higher linker decomposition compared to the freshly prepared samples was observed, with intact *pcl*-H_2_bdc-Urea linker to H_2_bdc-NH_2_ ratios decreasing from around 6:1 to 2:1.

For this proof-of-concept system, linker decomposition could be reduced through partial healing through additional growth steps, sample handling procedures at or near room temperature and through avoiding prolonged storage periods. Looking forward, the development of thermally more robust crosslinking units in *pre-connected linker* MOFs will provide a pathway to higher thermal stability in these modified MOFs.

**Figure S 6.** TG-MS curves of pcl-UiO-66-Urea with the MS ion current corresponding to CO_2_ shown. Decomposition of the urea crosslink is evident starting just above room temperature until 120 °C. Following the removal of the crosslinks, a crystalline UiO-66-NH_2_ framework persists, which then starts to decompose at approximately 300 °C. It is evident from the TG that sample handling at elevated temperatures is not possible while preserving the crosslink structure. Therefore, activating the framework under mild conditions is necessary.

**Figure S 7.** Argon sorption isotherms (87 K) of pcl-UiO-66-Urea after the initial synthesis step (orange), first growth step (red) and second growth step (brown). The linear (a) and logarithmic (b) isotherms are shown with closed symbols indicating the adsorption branch and open symbols indicating the desorption branch. A smaller degree of interparticular condensation is evident with increasing particle size from the lower uptake registered at relative pressures above 0.8. Furthermore, the increased slope of the seed crystal isotherm between *p*/p_0_ 0.2-0.7 is characteristic for mesoporosity arising from missing cluster and linker defects, which we propose are healed during subsequent growth steps.

**Figure S 8.** ^1^H NMR of digested *pcl*-UiO-66-Urea MOF after each growth step. Full spectra (a) and zoomed-in spectra of the aromatic linker region (b) with signal region of the linker decomposition product are shown. Signals appear shifted between different spectra due to slight differences in pH values of the digested solutions. Samples were digested after Soxhlet solvent exchange with DCM.

**Figure S 9.** Correlation between mean particle diameter and *pcl*-H_2_bdc-Urea:H_2_bdc-NH_2_ linker ratios, determined from ^1^H-NMR digests shown in Figure S 8. It is evident that lower linker decomposition is observed with larger particle sizes, which we attribute to defect healing and the increased particle bulk:surface ratio.

**Figure S 10.** Solvent stability of *pcl*-UiO-66-Urea. A large particle sample, stored for several months under ambient conditions (at room temperature), was submerged in various solvents for 3 d at room temperature. Powder X-ray diffractograms (a) prove the structural stability of the *pcl*-UiO-66-Urea framework under the same conditions typically used to test regular UiO-66. ^1^H-NMR spectra of the digests (b) show that the *pcl*-H_2_bdc-Urea linker (signal regions are highlighted in blue, the formic acid region has been removed for clarity) remains mostly intact across all tested conditions even after several months of storage (with the exception of pH 13, where the entire MOF degraded). Ratios of the intact linker to H_2_bdc-NH_2_, determined from NMR signal integrals (c) show no notable variance between the tested submersion conditions.

### S4.3. Electron crystallography

**Table S 1.** 3DED parameters, residuals and crystallographic data for kinematical refinement of UiO-66 at room temperature.

|  | UiO-66 at RT |
| --- | --- |
| **Crystal data** |  |
| Crystal system, space group | cubic, *F m*-3*m* |
| *a* (Å) | 20.7768(8) |
| *V* (Å³) | 8968.8(6) |
| Empirical formula | Zr_3_O_16_C_24_H_12_ |
| *Z* | 8 |
|  |  |
| **Data collection** |  |
| Radiation type | electrons |
| Wavelength (Å) | 0.019687 |
| Temperature (K) | 293 |
| No. 3DED data sets (used/total) | 2/2 |
| *α*_min_, *α*_max_ (deg.) (#1 / #2) | -40, 40 / -40, 40 |
| Angular speed (deg./s) | 0.88531 |
| Effective angular step (deg./frame) | 0.76187 |
| Exposure time per frame (ms) | 500 |
| Resolution (Å^-1^) | 1.3 |
| No. of total frames | 211 |
| Completeness (%) | 100 |
|  |  |
| **Kinematical structure refinement** |  |
| No. of used reflections (obs/all) | 424/577 |
| *R*_1_(obs)/*R*_1_(all), *wR*_1_(obs)/*wR*_1_(all) (%) | 20.3/22.0, 18.9/20.0 |
| goodness of fit (obs/all) | 3.1/2.8 |
| No. of refined parameters | 27 |

**Table S 2.** SerialPED parameters, residuals and crystallographic data for kinematical refinement of *pcl*-UiO-66-Urea and EtNO_2_@*pcl*-UiO-66-Urea.

|  | *pcl*-UiO-66-Urea at RT | EtNO_2_@*pcl*-UiO-66-Urea at RT |
| --- | --- | --- |
| **Crystal data** |  |  |
| Crystal system, space group | cubic, *F m*-3*m* | cubic, *F m*-3*m* |
| *a* (Å) | 20.7928(4) | 20.7753(6) |
| *V* (Å³) | 8989.6(3) | 8966.9(4) |
| Empirical formula | Zr_3_O_16_C_24_H_9_N_3_ | Zr_3_O_16_C_24_H_9_N_3_ |
| *Z* | 8 | 8 |
|  |  |  |
| **Data collection** |  |  |
| Radiation type | electrons | electrons |
| Wavelength (Å) | 0.025079 | 0.025079 |
| Temperature (K) | 293 | 293 |
| No. PED patterns (used/total) | 121/171 | 172/207 |
| Precession angle (deg.) | 0.88 | 0.98 |
| Exposure time per frame (ms) | 250 | 250 |
| Resolution (Å^-1^) | 1.25 | 1.25 |
| Completeness (%) | 100 | 100 |
|  |  |  |
| **Kinematical structure refinement** |  |  |
| No. of used reflections (obs/all) | 469/520 | 508/513 |
| *R*_1_(obs)/*R*_1_(all), *wR*_1_(obs)/*wR*_1_(all) (%) | 18.2/19.1, 19.6/19.7 | 19.0/19.1, 21.5/21.5 |
| goodness of fit (obs/all) | 6.1/5.8 | 7.0/7.0 |
| No. of refined parameters | 34 | 34 |

**Figure S 11.** Pawley fit of *pcl*-UiO-66-Urea and EtNO_2_@*pcl*-UiO-66-Urea samples.

### S4.4. Vapor sorption and reference MOF characterization

**Figure S 12.** Determination of Henry constants from nitroethane vapor sorption isotherms (298 K) of (a)UiO-66, (b)UiO-66-NH_2_ and (c) *pcl*-UiO-66-Urea.

**Figure S 13.** Powder diffractograms of UiO-66 (a, black) and UiO-66-NH_2_ (b, blue).

**Figure S 14.** Argon sorption isotherms (87 K) of UiO-66 (black) and UiO-66-NH_2_ (blue). Determined BET surface areas are 1212 m^2^g^-1^ and 975 m^2^g^-1^, respectively. The linear (a) and logarithmic (b) isotherms are shown with closed symbols indicating the adsorption branch and open symbols indicating the desorption branch.

**Figure S 15.** IR (a) and UV-Vis (b) spectra of *pcl*-UiO-66-Urea in comparison to reference MOFs. The wavenumber region corresponding to CO_2_ is removed for clarity in (a).

**Figure S 16.** Powder diffractograms (a) and ^1^H NMR (b) of digested MOFs (UiO-66: black; UiO-66-NH_2_: blue; *pcl*-UiO-66-Urea: brown) after nitroethane vapor sorption proving framework and linker intactness.

## S5. Structural considerations

### S5.1 Connection types in the UiO-66 structure

Regarding crosslink design, various possible bridging distances within the UiO-type framework have to be considered. To limit the number of potential crosslink configurations, we focused on the shortest distances within the tetrahedral and octahedral pores of the structure. As shown in Figure S 17(a) and (b), crosslinking directly across the centers of the tetrahedral and octahedral pores requires bridges capable of spanning C-C distances exceeding 10 Å. Such configuration will not result in periodic structures if the framework consists solely of bridged linkers, because the pore centers cannot accommodate the steric bulk of many crosslinks. Additionally, crosslinks with the required 12.3 Å to bridge the octahedral pores could also be introduced in the tetrahedral pores, if the crosslink is flexible enough to bend and shorten its C-C distance to 10.4 Å. For two linkers connected to the same IBU, assuming both linker carbon atoms closest to the IBU are bridged, the shortest C-C distances are across the square base area spanning the octahedral pore (7.6 Å) shown in Figure S 17(c) and across the trigonal pore windows (5.4 Å) shown in Figure S 17(d). For both shorter crosslink types, we predict the existence of periodic structures composed of entirely bridged linkers. For the crosslink type spanning the trigonal pore windows, three of the four pore windows of every second tetrahedral pore should be bridged by the crosslinks averaged across the entire structure. Mixed configurations are also possible; for example, one pore could feature two crosslinks across its windows while an adjacent pore features only one (discussed in detail in section S5.2 Connection pattern). Notably, unlike the crosslink type spanning across the square cross section of the octahedral pore, the trigonal window crosslink does not lie in the plane of the linker phenyl rings. We therefore hypothesize that crosslinks slightly shorter than 5.4 Å are compatible with the crosslinking type across the trigonal pore windows, as they could be accommodated through out-of-plane twisting of the phenyl rings.

**Figure S 17.** Shortest possible C-C crosslinking distances in the UiO-66 structure. It is assumed that the entire framework consists entirely of bridged linkers. (a) Crosslinking across the tetrahedral pore and (b) across the octahedral pore will not result in periodic structures, as the pores cannot accommodate multiple crosslinks bridging through the same pore center. In contrast, (c) Crosslinking across the square base area spanning up the octahedral pore and (d) across the trigonal pore windows (shared by the octahedral and tetrahedral pores) allow for the formation of periodic structures, as these shorter crosslinks do not cause steric hindrance. For the trigonal pore windows in (d), linkers can tilt out of the carboxylate plane toward one another to accommodate slightly shorter crosslinks.

### S5.2 Connection pattern across trigonal pore windows

The structures resulting from connecting linkers across the trigonal pore windows are very diverse, due to the vast number of possible connection patterns. This complexity can be best understood by dividing the unit cell into the eight tetrahedral pores. Within each pore, there are four possible types of configurations, differing by their number of crosslinks. With six total linkers within one tetrahedron, it is possible to have 0, 1, 2 or 3 crosslinks, shown in Figure S 18. Notably neither the orientation of these building blocks nor the internal connection pattern is fixed.

The challenge in identifying possible patterns lies in how these building blocks connect to form a UiO-66 type unit cell. In this structure, linkers are shared between adjacent tetrahedra. Each linker must be connected to exactly one other linker via a crosslink, which significantly constrains the possible configuration and adds immense complexity.

**Figure S 18.** The only possible numbers of crosslinks in tetrahedral pores. Note that while these represent the possible total number of crosslinks per pore, their location (which linkers are connected) and their relative orientation can vary.

To identify all possible crosslink patterns within the UiO-66 system for trigonal window bridging, we developed a Python script. The algorithm uses idealized coordinates for the mid-points of the linkers to calculate an adjacency matrix. This matrix is filtered to include only the shortest connection paths, thereby enforcing connections across the trigonal pore windows. Afterwards, a recursive search using a minimum weight algorithm is performed to identify graphs across those points where no two points share a common vertex. Whenever a valid graph is identified, one of its edges is removed and the search repeated. Once no further valid graphs can be found, octahedral symmetry is applied to identify identical connection patterns.

Using this process, a total number of 34174 connection patterns was identified. These patterns can be represented as combinations of 8 tetrahedral pores with 0, 1, 2 or 3 crosslinks.

Table S 3 shows the different combinations identified using the script and the corresponding number of configurations for each combination.

**Table S 3.** Possible combinations of tetrahedrons with 0-3 crosslinks across the trigonal windows that can assemble into a *pcl-*UiO-66 structure. In total, a unit cell of *pcl*-UiO-66 contains 8 tetrahedral pores. The table presents the identified combinations and the number of distinct configurations corresponding to each combination.

| Combination | Number of configurations |
| --- | --- |
| 4x1, 4x2 | 15286 |
| 1x0, 3x1, 3x2, 1x3 | 6320 |
| 1x0, 2x1, 5x2 | 5888 |
| 5x1, 2x2, 1x3 | 3072 |
| 2x0, 2x1, 2x2, 2x3 | 1040 |
| 2x0, 1x1, 4x2, 1x3 | 1024 |
| 2x0, 6x2 | 664 |
| 3x0, 3x2, 2x3 | 344 |
| 4x0, 4x3 | 186 |

## S6. Additional simulation data

### S6.1 Additional trajectory snapshots

**Figure S 19.** Trajectory snapshots of nitroethane in UiO-66-NH_2_. The current frame is marked by an orange sphere, the blue spheres show the locations of previous frames. The model is simplified for better visibility with the black rods corresponding to bdc-NH­_2_-type linkers and the purple spheres corresponding to the Zr-Clusters.

**Figure S 20.** Trajectory snapshots of propane in UiO-66-NH_2_. The current frame is marked by an orange sphere, the blue spheres show the locations of previous frames. The model is simplified for better visibility with the black rods corresponding to bdc-NH­_2_-type linkers and the purple spheres corresponding to the Zr-Clusters.

**Figure S 21.** Trajectory snapshots of nitroethane in *pcl-*UiO-66-Urea. The current frame is marked by an orange sphere, the blue spheres show the locations of previous frames. The model is simplified for better visibility with the black rods corresponding to bdc-type linkers connected via Urea groups (red rods) and the purple spheres corresponding to the Zr-Clusters.

**Figure S 22.** Trajectory snapshots of propane in *pcl-*UiO-66-Urea. The current frame is marked by an orange sphere, the blue spheres show the locations of previous frames. The model is simplified for better visibility with the black rods corresponding to bdc-type linkers connected via Urea groups (red rods) and the purple spheres corresponding to the Zr-Clusters.

### S6.2 Additional pore mobility analysis

**Figure S 23.** Graphs showing the location of the propane molecule inside the *pcl*-UiO-66-Urea during the MD simulation, classified by pore type.

**Figure S 24.** Graphs showing the location of the nitroethane molecule inside the *pcl*-UiO-66-Urea during the MD simulation, classified by pore type.

**Figure S 25.** Graphs showing the location of the nitroethane molecule inside the UiO-66-NH­_2_ during the MD simulation, classified by pore type.

**Figure S 26.** Graphs showing the location of the propane molecule inside the UiO-66-NH­_2_ during the MD simulation, classified by pore type.

### S6.3 Binding energy of DMDNB and nitroethane

Table S 4 shows binding energies *E_bind_* calculated from structures optimized using force field methods. To reduce computational cost, the entire MOF was reduced to a single functionalized pore. The connection pattern is the same as shown in Figure S 18 (3). An atomistic representation of this pore can be seen in Figure 5 in the main manuscript. The IBUs were capped using formic acid. A single probe molecule was placed inside the investigated pore for the global minimum structure using a Quench MD (*NVT*, 300 K 50 ns, 1000 optimized structures). DMDNB displays a higher affinity for the pore compared to nitroethane.

**Table S 4** Binding energies *E_bind_* for DMDNB and nitroethane inside a functionalized pore. These energies were calculated by subtracting the total energy of the empty pore *E_pore_,* as well as the total energy of the isolated molecule *E_mol_* from the total energy of the filled pore *E_pore+mol_*.

| Molecule | DMDNB | Nitroethane |
| --- | --- | --- |
| *E_pore+mol_* / kcal/mol | −4088.82 | −4101.95 |
| *E_pore_* / kcal/mol | −4047.62 | |
| *E_mol_* / kcal/mol | 27.434 | 5.258 |
| Binding energy / kcal/mol | −68.634 | −59.588 |

# References

[1]	P. Roy, A. Schaate, P. Behrens, A. Godt, "Post-synthetic modification of Zr-metal-organic frameworks through cycloaddition reactions," *Chem. - Eur. J.* **18** (2012): 6979.

[2]	M. Santucci, U. Kolb, "Evaluation of the reproducibility and crystal tracking precision of TEM goniometers in tomography experiments," *Ultramicroscopy* **282** (2026): 114308.

[3]	S. Plana-Ruiz, J. Portillo, S. Estradé, F. Peiró, U. Kolb, S. Nicolopoulos, "Quasi-parallel precession diffraction: Alignment method for scanning transmission electron microscopes," *Ultramicroscopy* **193** (2018): 39.

[4]	S. Plana-Ruiz, P. Lu, G. Ummethala, R. E. Dunin-Borkowski, "On the use of beam precession for serial electron crystallography," *J. Appl. Crystallogr.* **58** (2025): 1249.

[5]	L. Palatinus, P. Brázda, M. Jelínek, J. Hrdá, G. Steciuk, M. Klementová, "Specifics of the data processing of precession electron diffraction tomography data and their implementation in the program PETS2.0," *Acta Crystallogr., Sect. B:Struct. Sci.* **75** (2019): 512.

[6]	E. F. Rauch, L. Dupuy, "Rapid Diffraction Patterns identification through template matching," *Arch. Metall. Mater.* **50** (2005): 87.

[7]	V. Petříček, L. Palatinus, J. Plášil, M. Dušek, "Jana2020 – a new version of the crystallographic computing system Jana," *Z. Kristallogr. - Cryst. Mater.* **238** (2023): 271.

[8]	G. S. Pawley, "Unit-cell refinement from powder diffraction scans," *J. Appl. Crystallogr.* **14** (1981): 357.

[9]	W. I. F. David, K. Shankland, J. van de Streek, E. Pidcock, W. D. S. Motherwell, J. C. Cole, "DASH : a program for crystal structure determination from powder diffraction data," *J. Appl. Crystallogr.* **39** (2006): 910.

[10]	L. Palatinus, G. Chapuis, "SUPERFLIP – a computer program for the solution of crystal structures by charge flipping in arbitrary dimensions," *J. Appl. Crystallogr.* **40** (2007): 786.

[11]	E. A. Hall, L. R. Redfern, M. H. Wang, K. A. Scheidt, "Lewis Acid Activation of a Hydrogen Bond Donor Metal–Organic Framework for Catalysis," *ACS Catal.* **6** (2016): 3248.

[12]	Didier Cordier and Pierre R. Coulet, "Metal-ion Complexation by a New Urea Macrocyclophane," *J. Chem. Soc., Perkin Trans. 1 (1972-1999)* (1994).

[13]	S. Dai, A. Tissot, C. Serre, "Metal-Organic Frameworks: From Ambient Green Synthesis to Applications," *Bull. Chem. Soc. Jpn.* **94** (2021): 2623.

[14]	K. M. Zwoliński, P. Nowak, M. J. Chmielewski, "Towards multifunctional MOFs--transforming a side reaction into a post-synthetic protection/deprotection method," *Chem. Commun. (Cambridge, U. K.)* **51** (2015): 10030.

[15]	W. Morris, C. J. Doonan, O. M. Yaghi, "Postsynthetic modification of a metal-organic framework for stabilization of a hemiaminal and ammonia uptake," *Inorg. Chem.* **50** (2011): 6853.

[16]	C. Avci-Camur, J. Perez-Carvajal, I. Imaz, D. Maspoch, "Metal Acetylacetonates as a Source of Metals for Aqueous Synthesis of Metal–Organic Frameworks," *ACS Sustainable Chem. Eng.* **6** (2018): 14554.
